# College and Hospital Notes

**Published:** 1906-09

**Authors:** 


### COLLEGE AND HOSPITAL NOTES.

The Medical Department of the University of Buffalo will open its sixty-first annual session September 24, 1906, and continue thirty-four weeks. The advanced matriculation and registration list indicates a large attendance for the academic year. Dr. James E. King, adjunct professor of obstetrics, will deliver the opening lecture of the course in Alumni Hall, Monday evening, September 24, at eight o'clock.

Great Military Hospital.—The War Department is preparing to build near Washington, on Brightwood Road, within the district limits, a large army general hospital, which will be the best equipped institution of its kind in the world. For months the army surgeons have been engaged in looking up literature on the subject and inspecting hospitals in all parts of the country. They have also obtained much information from abroad. The best ideas which prevail anywhere will be used in the new hospital, which will cost a little less than $500,000. To this institution army officers and enlisted men from all parts of the country and the Philippines will be sent when they are in need of special treatment. There is a general hospital now at Washington Barracks, but it is not up to date and is not large enough to meet all the demands on it.

